# Contrasting pattern of subtelomeric satellites in the Cannabaceae family

**DOI:** 10.3389/fpls.2025.1631369

**Published:** 2025-08-19

**Authors:** Lucie Horáková, Václav Bačovský, Radim Čegan, Bohuslav Janoušek, Josef Patzak, Roman Hobza

**Affiliations:** ^1^ Department of Plant Developmental Genetics, Institute of Biophysics of the Czech Academy of Sciences, Brno, Czechia; ^2^ Department of Experimental Biology, Faculty of Science, Masaryk University, Brno, Czechia; ^3^ Hop Research Institute Co. Ltd., Žatec, Czechia

**Keywords:** subtelomeric repeats, *Humulus*, satellite divergence, phylogenetics, metaphase chromosomes

## Abstract

**Introduction:**

Satellite DNA (satDNA) is a rapidly evolving component of plant genomes, typically found in (peri)centromeric, (sub)telomeric, and other heterochromatic regions. Due to their variability and species- or population-specific distribution, satDNA serves as valuable cytogenetic markers for studying chromosomal rearrangements and karyotype evolution among closely related species. Previous studies have identified species-specific subtelomeric repeats CS-1 in *Cannabis sativa*, HSR1 in *Humulus lupulus*, and HJSR in *Humulus japonicus*. These satellites have been used to differentiate sex chromosomes from autosomes, however, their evolutionary origins, sequence variation and conservation pattern across related species remain largely unexplored.

**Methods:**

In this study, we analyze sequence similarity among these satellites and assess their interspecific chromosomal localization using fluorescence in situ hybridization (FISH).

**Results:**

Our results reveal that the HSR1 and HJSR satellites are shared across all studied species, suggesting their common origin from a shared pool of satDNA in their common ancestor. In contrast, the CS-1 satellite exhibits higher sequence divergence.

**Discussion:**

Although all three satellites are predominantly localized in subtelomeric regions, we identified species-specific exceptions. These findings provide new insight into the evolutionary dynamics of satDNA within the Cannabaceae family and offer further support for the divergence of *Humulus* species.

## 1 Introduction

Plant genomes are predominantly composed of repetitive DNA, which can account for up to 85% of the total genome (Novák et al., 2020). This repetitive fraction is broadly classified into two main categories: dispersed (transposable elements) and tandem repeats (satellite DNA = satDNA). SatDNA is composed of monomer sequences organized in long tandem arrays, which tend to accumulate primarily in chromosomal subdomains characterized by a condensed, heterochromatic organization - such as (peri)centromeres and (sub)telomeres (Garrido-Ramos, 2015). SatDNA represents one of the most rapidly evolving components of eukaryotic genomes, varying among related species in (i) nucleotide sequence, (ii) copy number, (iii) length of monomer subunits, and (iv) chromosome localization (Plohl et al., 2008; Lower et al., 2018). SatDNA is further enriched in specialized chromosomes, such as B chromosomes or sex chromosomes. In the latter, enrichment is associated with low or suppressed recombination, which facilitates the expansion of satellite repeats and the accumulation of transposable elements (TEs) in the non-recombining regions of the Y or W chromosome (Palacios-Gimenez et al., 2017; Jesionek et al., 2021). In addition to their dynamic nature, large satDNA arrays have a role in chromosome evolution and segregation, highlighting their functional significance (Plohl et al., 2012). Due to their repetitive structure, satDNA is a robust and powerful tool for cytogenetic studies. It enables detailed analysis of individual chromosomes (Hobza et al., 2024), chromosomal rearrangements, and evolutionary dynamics among related species (Jagannathan et al., 2017; Schmidt et al., 2019, 2024). According to the library hypothesis, related species share a set of satellite sequences that exhibit certain level of sequence divergence and variation in abundance. The differential amplification of specific satellite sequences leads to unique satDNA collections in related species, which may display species- or population-specific profiles (Fry et al., 1973; Mestrovic et al., 1998; Plohl et al., 2012). This hypothesis has been extensively studied in various plants (Koukalova et al., 2010; Belyayev et al., 2019, 2020), fish (Utsunomia et al., 2017; Goes et al., 2022), and other eukaryotic organisms (Mestrovic et al., 1998; Camacho et al., 2022). Furthermore, satDNA monomers are typically homogenized within a species as a consequence of concerted evolution, a process that maintains sequence uniformity within a species while promoting interspecies divergence (Waye and Willard, 1989; Durfy and Willard, 1990; Wei et al., 2014).

The Cannabaceae family comprises the genera *Cannabis* and *Humulus*, along with eight additional genera widely distributed across tropical and temperate regions (Yang et al., 2013; Jin et al., 2020). The genus *Cannabis*, thought to have originated in East Asia, includes a diploid species, *Cannabis sativa* (Lapierre et al., 2023), which is cultivated for fiber, oil, and as a source of therapeutic compounds (Small, 2015). The sister genus *Humulus* consists of three species: *Humulus lupulus* L., *H*. *japonicus* Siebold & Zucc. (synonym *H*. *scandens* (Lour.) Merr.), and the Chinese endemic *H*. *yunnanensis* Hu (Ling and Zhang, 2019). *Humulus lupulus*, commonly known as hop, is a key ingredient in beer brewing. Together with *H*. *japonicus*, it produces unique secondary metabolites with significant therapeutic potential (Yu et al., 2007; Zanoli and Zavatti, 2008; Ryu et al., 2017; Jiang et al., 2018). The divergence between *C*. *sativa* and *H*. *lupulus* is estimated to have occurred between 16 and 27.8 million years ago (mya; McPartland, 2018; Jin et al., 2020; Prentout et al., 2021; Padgitt-Cobb et al., 2023). In contrast, *H*. *lupulus* and *H*. *japonicus* diverged more recently, approximately 3.7 – 10.7 mya (Murakami, 2000; Murakami et al., 2006; Jin et al., 2020). Members of both *Cannabis* and *Humulus* genera exhibit dioecy, but some individuals occasionally develop as monoecious. It is hypothesized that dioecy evolved prior to the divergence of these two genera, at least 21–25 mya (Prentout et al., 2021; Akagi et al., 2025).

The three species within the *Cannabis* and *Humulus* genera, namely *C*. *sativa*, *H*. *lupulus*, and *H*. *japonicus*, exhibit significant differences in genome size, ranging from 808 Mb in *C*. *sativa* (Gao et al., 2020) to 1.8 Gb in *H*. *japonicus* (Grabowska-Joachimiak et al., 2006) and 2.8 Gb in *H*. *lupulus* (Padgitt-Cobb et al., 2023). A substantial proportion of these genomes is composed of repetitive DNA: 64-74.8% in *C*. *sativa* (Gao et al., 2020; Pisupati et al., 2018; Lynch et al., 2025), 61.3-64.5% in *H*. *lupulus* (Pisupati et al., 2018; Padgitt-Cobb et al., 2023; Horáková et al., 2025), and 66.8% in *H*. *japonicus* (Zhang et al., 2023). Consistent with patterns observed in plant genomes (Feschotte et al., 2002), long terminal repeat retrotransposons (LTR-RTs) represent the most abundant class of repetitive DNA across *Cannabis* and *Humulus* species (Pisupati et al., 2018; Zhang et al., 2023; Lynch et al., 2025). A burst of LTR-RTs proliferation may have contributed to the enlargement and structural rearrangement of the X chromosomes in both *Humulus* species (Akagi et al., 2025). Interestingly, the *H*. *japonicus* has undergone an X-autosome fusion involving autosome 3, leading to the formation of neo-X chromosome arm and neo-Y chromosomes (Akagi et al., 2025). In contrast, the accumulation of Ty1/*Copia* solo-LTRs has predominantly shaped the Y chromosome in *C*. *sativa* (Lynch et al., 2025). Meanwhile, satDNA constitutes only a small fraction of the repetitive DNA in *Humulus* species, accounting for 0.3-2% of the genome (Zhang et al., 2023; Horáková et al., 2025). Recent studies using long-read PacBio sequencing and advanced bioinformatics tools have characterized tandem repeat families in *H*. *lupulus* (Easterling et al., 2020) and the most abundant DNA repeats in *H*. *japonicus* (Zhang et al., 2023). To date, the most valuable cytogenetic markers for identifying autosomes and sex chromosome in *Cannabis* and *Humulus* species include three members: CS-1 (*Cannabis sativa* 1; Divashuk et al., 2014), HSR1 (*Humulus* subtelomeric repeat 1; Divashuk et al., 2011), and HJSR (*Humulus japonicus* subtelomeric repeat; Alexandrov et al., 2012). Both subtelomeric HSR1 and HJSR satellites were originally characterized through restriction digestion using the *Kpn*I endonuclease (Divashuk et al., 2011; Alexandrov et al., 2012), whereas CS-1 was later derived from HSR1 as a repeat with low homology to the HSR1 satellite (Divashuk et al., 2014). This may suggest common origin for both repeats, classifying them into one superfamily, similar to patterns observed in other plats species such as *Rumex acetosa* (Navajas-Pérez et al., 2006; Cuñado et al., 2007; Navajas-Pérez et al., 2009b; Quesada Del Bosque et al., 2011). Despite these three satellites were identified independently in *Humulus* and *Cannabis* species, they exhibit remarkable similarities, including their subtelomeric localization. However, the origin and evolutionary dynamics of these large satellite arrays remain poorly understood, particularly compared to those described in other plant species. In this study, we isolated and compared major satellites CS-1 (*C*. *sativa*-specific), HSR1 (*H*. *lupulus*-specific), and HJSR (*H*. *japonicus*-specific). We identified their interspecific chromosomal localization using fluorescent *in situ* hybridization (FISH) in both sexes. To quantify their genome abundance, sequence similarity, and intraspecific variability, we combined short Illumina reads with bioinformatic analyses, and molecular cloning and phylogenic analysis within each species. Given the parallel origin of CS-1, HSR1, and HJSR, we aimed to compare (i) the genetic differentiation of the HSR1 and HJSR satellites in the context of *H*. *japonicus* speciation, and (ii) the origin and divergence of this subtelomeric satellite family. We also discussed the sequence similarity and chromosomal distribution of the HSR1 and CS-1 satellites to gain insight into their evolutionary relationship.

## 2 Materials and methods

### 2.1 Plant material

Female *H*. *lupulus* cv Saaz hop (Osvald’s clone 72) and male *H*. *lupulus* Lib male (15181) (2n = 18 + XX/XY) were provided by the Hop Research Institute Co. Ltd. in Žatec (Czech Republic). Female and male *H*. *japonicus* (2n = 14 + XX/XY_1_Y_2_) and *C*. *sativa* cv Kompolti (2n = 18 + XX/XY) plants were grown from seeds obtained from W. Legutko (Poland) and SEMO a.s. (Czech Republic), respectively. All plants were grown in a greenhouse under controlled conditions (16h daylight/8h dark photoperiod) at the Department of Plant Developmental Genetics in Brno (Czech Republic). The sex of *H*. *japonicus* plants was determined based on floral morphology and chromosome number. In *Cannabis sativa* and *H*. *lupulus*, sex was determined using PCR with male-specific molecular markers: MADC2 for *C*. *sativa* (Mandolino et al., 1999) and OPJ9 for *H*. *lupulus* (Polley et al., 1997). Both male-specific markers were amplified using Taq polymerase (Top Bio) according to the manufacturer’s instructions (for primers, see **Supplementary Table S1**). PCR cycling conditions followed protocols described by Razumova et al. (2016) and Patzak et al. (2002). Male and female plants of each species were selected for further analysis based on the PCR results (**Supplementary Figure S1**).

### 2.2 Isolation of DNA and Low-coverage genome sequencing

Genomic DNA was extracted from young leaves of male *C*. *sativa*, *H*. *lupulus*, and *H*. *japonicus*, using the NucleoSpin Plant II (Macherey-Nagel GmbH and Co. KG., Germany), following the manufacturer’s protocol. Libraries were prepared using the NEBNext^®^ Ultra™ II DNA Library Prep Kit. Genomic DNA from male plants of *C*. *sativa* and *H*. *japonicus* was low-coverage sequenced using an Illumina MiSeq sequencer, generating 300 bp paired-end reads at the Centre of Plant Structural and Functional Genomics (Olomouc, Czech Republic). Additionally, we used the library of *H*. *lupulus* described in Horáková et al. (2025).

### 2.3 Analysis of repeats using Repeat Explorer

The FastQC tool (available at http://www.bioinformatics.babraham.ac.uk/projects/fastqc) was used to assess the quality of sequencing reads. Furthermore, reads were pre-processed based on quality (Q30) with subsequent adaptor trimming, filtering out short or unpaired sequences, and all reads were trimmed to a uniform length of 200 bp using Trimmomatic 0.32 (Bolger et al., 2014). To identify repetitive DNA composition and tandem repeats, these datasets were analyzed using RepeatExplorer2 (Novák et al., 2013, 2020) and Tandem Repeat Analyzer (TAREAN) pipelines (Novák et al., 2017). The clustering of randomly selected 2 x 500–000 reads was performed by default setting.

### 2.4 Ligation of PCR products and cloning

The major satellites, including Cl5 (CS-1), Cl75 (HSR1), and Cl57 (HJSR) were amplified by PCR using specific primers (**Supplementary Table S1**). Primers were designed in Geneious Prime (version 2023.1.1) based on the consensus sequences generated by the TAREAN pipeline. PCR amplification was performed using Q5 High-Fidelity DNA polymerase (M0491S; NEB) according to the manufacturer’s instructions. The cycling conditions were as follows: 95°C for 4 min followed by 35 cycles of 94°C for 30 s, 52 - 57°C for 35 s, 72°C for 30 s, and a final extension step at 72°C for 10 min. The annealing temperature was optimized for each primer pair. Selected units for each satellite (**Supplementary Figure S2**) were extracted from agarose gel and purified using the QIAquick Gel Extraction Kit (QIAGEN GmbH - Hilden, Germany).

The purified fragments were individually cloned into the pJET1.2 plasmid vector using the CloneJET PCR Cloning Kit (K1231; ThermoFisher), following the manufacturer’s instructions. Ligation reactions were incubated overnight at 16°C and subsequently desalted. Competent *E*. *coli* cells were transformed by electroporation using a MicroPulser Electroporator (Bio-Rad). After 30-minute incubation, transformed cells were plated onto LB medium containing ampicillin (100 mg/L) and incubated overnight at 37°C. The next day, colony PCR was performed using pJET1.2-specific primers to identify positive clones. Individual bacterial colonies were used as templates. From positive PCR products, residual primers and PCR dNTPs were removed using ExoSAP reaction. PCR amplicons were sequenced in Macrogen (Amsterdam, Netherlands).

### 2.5 DNA probes preparation

Ribosomal DNA (5S and 45S rDNA) was amplified by PCR using specific primers (**Supplementary Table S1**). The primers were designed in Geneious Prime (version 2023.1.) based on the sequences from GenBank database (accession numbers: MN537579 (Easterling et al., 2020) and AF223066.1). PCR amplification was performed in a 20 µl reaction mixture containing 1x PCR buffer, 0.0001 M dNTPs, 0.0001 M of each primer, 0.5 U Taq polymerase (Top Bio), and 10–15 ng of template DNA. The PCR cycling conditions were as follows: initial denaturation at 95°C for 4 min, 35 cycles at 94°C for 30 s, 55°C for 35 s, 72°C for 30 s, followed by a final extension at 72°C for 10 min. The telomeric repeat sequence (Arabidopsis-type, TTTAGGG) was amplified without template DNA according to Ljdo et al. (1991). PCR cycling conditions were: first denaturation at 94°C for 1 min, 10 cycles of 94°C for 1 min, 55°C for 30 s, and 72°C for 1 min, followed by 30 cycles of 94°C for 1 min, 60°C for 30 s, 72°C for 90 s, and final extension step at 72°C for 5 min.

PCR products were separated on a 1% agarose gel with EtBr staining and purified using the QIAquick PCR Purification Kit (28104; QIAGEN) according to the manufacturer’s instructions. Purified PCR products (1µg) of each satellite and species and 5S, 45S rDNA, and telomere obtained as described above, were labeled using Nick Translation Labelling kits (Jena Bioscience, Germany) with Atto488 NT (PP-305L-488), Atto550 NT (PP-305L-550), and Cy5 (PP-305L-647N), following the manufacturer’s instructions. Labeling reactions were incubated at 15°C for 90 min. The labeled products were analyzed on a 1% agarose gel with EtBr staining. Reactions of well-labeled DNA probes were stopped by adding 0.5M EDTA and incubating at 85°C. The labeled DNA probes were then used directly in the hybridization mixture for FISH.

### 2.6 Mitotic chromosome preparation and Fluorescence *in situ* hybridization

Mitotic chromosomes were prepared from young leaves (2–5 mm in length) of *C*. *sativa*, *H*. *lupulus*, and *H*. *japonicus* as described in Horáková et al. (2025). FISH was performed on mitotic metaphase chromosomes according to Sacchi et al. (2024) under two stringency conditions (77% and 68%; **Supplementary Table S2**). FISH with 77% stringency was used to confirm the subtelomeric localization of CS-1 (Cl5) in *C*. *sativa*, HSR1 (Cl75) in *H*. *lupulus*, HJSR (Cl57) in *H*. *japonicus*, and 45S rDNA. Low-stringency FISH (68%) was used to determine the interspecific localization of satellites. Chromosomes were counterstained with 4´,6´-diamidino-2-phenylindole (DAPI) in Vectashield Antifade Mounting Medium. Images were captured using an Olympus AX70 epifluorescence microscope equipped with a CCD camera and processed using Adobe Photoshop. FISH experiments were performed in triplicate, with at least ten metaphases analyzed per experiment.

### 2.7 Phylogenetic analysis of subtelomeric satellites

The sequences were aligned in MAFFT in two rounds. At first, the adjusted direction option was used to ensure the correct orientation of sequences. The alignment was further improved in MAFFT using an iterative refinement method incorporating local pairwise alignment (mafft-linsi). The phylogenetic tree was constructed using maximum-likelihood approach using IQ-TREE. The substitution model (HKY+F+G4) was chosen in IQ-TREE based on the BIC criterion. The support values were obtained using the ultrafast bootstrap method (with 1000 replicates). To obtain sequence logos for each satellite-species combination, we mapped the reads from a given species to the reference, which was represented by a 60% consensus sequence prepared from sequenced clones of PCR products obtained in the species in which that satellite was originally described (for primers, see **Supplementary Table S1**). In several satellite-species combinations, low-coverage sequencing did not allow sufficient coverage of the reference, so the sequence logo was prepared based on the cloned PCR products. DNA reads were trimmed using Trimmomatic (Bolger et al., 2014) and mapped to the reference using BWA-MEM (Li, 2013). SAM files were converted to fasta alignments using a simple Python script (sam2fasta.py; https://sourceforge.net/projects/sam2fasta/). Sequence logos were generated from fasta alignments using a locally installed version of the Weblogo 3 program (Crooks et al., 2004) with sequence-type set to ‘dna’.

## 3 Results

### 3.1 Analysis of repeat composition in *Cannabis* and *Humulus* species

Repeatome analysis revealed major satellites candidates in *Cannabis* and H*umulus* species. Specifically, Cl5 (CS-1) in *C*. *sativa*, Cl75 (HSR1) in *H*. *lupulus*, and Cl57 (HJSR) in *H*. *japonicus*. We extracted consensus sequence for each repeat and their abundance in the genomes based on sequencing data (**Supplementary Table S3**). These major satellites - Cl5 (CS-1), Cl75 (HSR1), and Cl57 (HJSR) show high sequence similarity to previously described sequences in GenBank: CS-1 (JX402748.2; Divashuk et al., 2014), HSR1 (*Humulus* subtelomeric repeat 1, GU831574.1; Divashuk et al., 2011), HJSR (*Humulus japonicus* subtelomeric repeat, GU831573.1; Alexandrov et al., 2012). For clarity and consistency, we refer to these repeats throughout the text by their established GenBank names: CS-1, HSR1, and HJSR, respectively. The monomer unit lengths for these satellites range from 370 bp for CS-1, 380 bp for HJSR, to 383 bp for HSR1. Sequence comparison revealed relatively higher sequence similarity between HSR1 and CS-1 (58.82%) and HSR1 and HJSR (65.05%; **Supplementary Figure S3**).

### 3.2 Comparison of major satellite distribution among the related species

**Figure 1:**
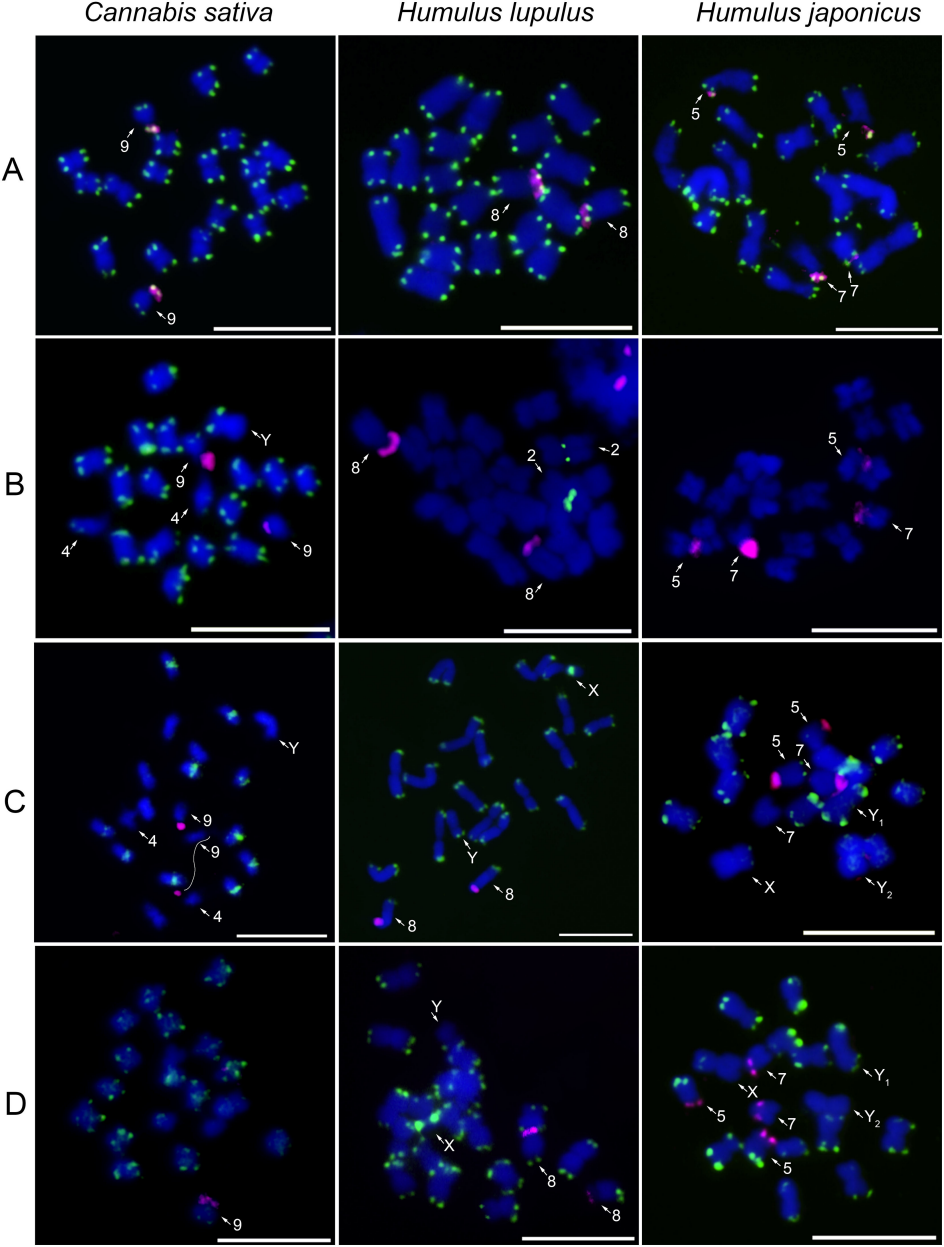
Chromosomal distribution of major satellites in studied species. The localization of **(A)** the telomeric sequence motif TTTAGGG (green), **(B)** the CS-1 satellite (green), **(C)** the HSR1 satellite (green), **(D)** the HJSR satellite (green), and 45S rDNA (magenta) on male mitotic metaphase chromosomes of *Cannabis sativa*, *Humulus lupulus*, and *H. aponicus*. Note the reduced number of CS-1 positive regions in *H. lupulus* and the absence of this satellite in *H. japonicus* **(B)**. Interestingly, HSR1 is localized in the (peri)centromeres in *C. sativa*, while it displays a subtelomeric position in both *H. lupulus* and *H. japonicus* **(C)**. The identification and numbering of individual chromosomes follow the system previously established and described by Kim et al. (2008); Divashuk et al. (2011, 2014); Alexandrov et al. (2012); Akagi et al. (2025). Mitotic chromosomes were counterstained with DAPI. Arrows indicate different autosomes and sex chromosomes. Scale bar = 10 µm.

To analyze the interspecific distribution of major satellites, CS-1, HSR1, and HJSR, we performed FISH on metaphase chromosomes of *C*. *sativa*, *H*. *lupulus*, and *H*. *japonicus* (**Figure 1**; **Table 1**). Additionally, *Arabidopsi*s-type telomeric (TTTAGGG) and 45S rDNA probes were used to label chromosome ends and enable the identification of previously described chromosomes (**Figure 1A**). Telomeric signals are present at the terminal regions of all chromosomes, without any interstitial telomeric signals (**Figure 1A**; **Supplementary Figure S4A**). The distribution of 45S rDNA varies among studied species, being localized on chromosome 9 in *C*. *sativa*, chromosome 8 in *H*. *lupulus*, and on two pairs of autosomes, specifically on chromosomes 5 and 7 in *H*. *japonicus* (**Figure 1A**). Chromosome nomenclature in *H*. *lupulus* is based on the pseudomolecule assembly by Akagi et al. (2025) with chromosomes numbered according to their size, from largest to smallest. Whereas in *C*. *sativa* and *H*. *japonicus*, it follows the systems established by Kim et al. (2008); Alexandrov et al. (2012), and Divashuk et al. (2014).

**Table 1:** Analyzed tandem repeats in *C*. *sativa*, *H*. *lupulus*, *H*. *japonicus*, the monomer unit size and its chromosomal distribution.

Satellite	Reference	Size (bp)	Chromosomal localization		
			*C*. *sativa*	*H*. *lupulus*	*H*. *japonicus*
CS-1	PRJEB81858	370	**+/subtelomere**	+/(peri)centromere	+/subtelomere
HSR1	PRJEB81858	383	+/(peri)centromere	**+/subtelomere, pericentromere (X chromosome)**	+/subtelomere
HJSR	PRJEB81858	380	–	+/subtelomere	**+/subtelomere**

+ Presence of satellite in species, - absence of satellite in species; Consensual sequences are listed in **Supplementary Data sheet 1**.

Bold values indicate the positions of major satellites in the species where they were originally identified

We observed chromosome-specific differences in the localization of all three studied satellites.

In *Cannabis sativa*, CS-1 is predominantly localized in the subtelomeric regions of both arms of all autosomes, except for the q-arm of chromosome 4 and the p-arm of chromosome 9, the latter of which possess 45S rDNA. Notably, the Y chromosome lacks CS-1 on the q-arm (**Figure 1B**). This distinct distribution of CS-1, combined with the Y chromosome size and DAPI banding pattern, enables its precise identification (**Figures 1B, C**). However, the identification of the X chromosome is still difficult, based solely on CS-1 distribution and factors mentioned above (**Figures 1B, C**). In *Humulus lupulus*, the CS-1 satellite is localized in the (peri)centromeric region of chromosome 2 (**Figure 1B**) while 5S rDNA is simultaneously detected in its subtelomeric region (**Supplementary Figure S5**). Although the CS-1 satellite was amplified with the designed primers in *H*. *japonicus* (**Supplementary Figure S2**), we did not observe any clear signals on metaphase chromosomes of either sex (**Figure 1B** and **Supplementary Figure S4B**), presumably resulting from its low abundance on chromosomes and in the genome.

The *Humulus lupulus*-specific repeat, HSR1 satellite, is localized in the (peri)centromeric regions of ten chromosomes in males (**Figure 1C**) and eleven chromosomes in females *C*. *sativa* (**Supplementary Figure S4C**). This structural chromosomal heterozygosity may indicate a hybrid origin of the female *C*. *sativa* cv Kompolti or suggest the presence of HSR1 on one of the X chromosomes. Simultaneous hybridization of 45S rDNA, HSR1, and CS-1 satellites shows that HSR1 is absent from chromosomes 4, 9, and Y (**Supplementary Figure S6**). In contrast, the HSR1 satellite is localized in the subtelomeric regions of almost all autosomes of *H*. *lupulus*, except for the p-arm of chromosome 8, which carries 45S rDNA, the p-arm of X chromosome, and the q-arm of chromosome Y (**Figure 1C**). Notably, the X chromosome exhibits a strong HSR1 signal in the pericentromeric region of its p-arm. In *Humulus japonicus*, the HSR1 probe is distributed in the subtelomeric regions of almost all chromosomes (**Figure 1C**), showing similarities to the HJSR probe (**Figure 1D**). However, HSR loci on one chromosome pair and the chromosome Y_2_ are absent or underrepresented.

**Figure 2:**
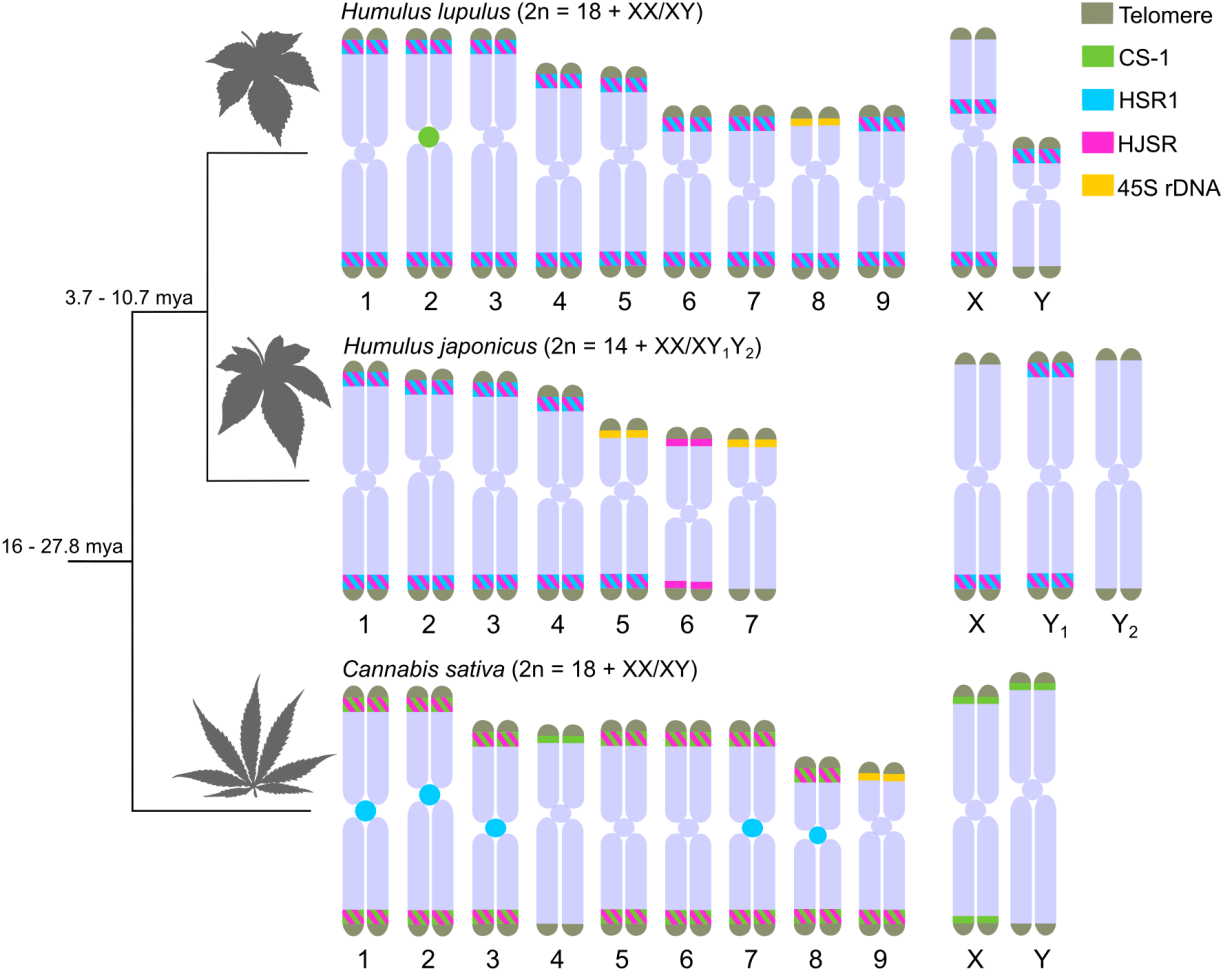
Idiograms illustrating chromosomal distribution of major satellites in *Humulus lupulus*, *H*. *japonicus*, and *Cannabis sativa*. The positions of telomere (grey), CS-1 (green), HSR1 (cyan), HJSR (magenta), and 45S rDNA (yellow) repeats were derived based on the FISH physical localization (**Figure 1** and **Supplementary Figures S5**-**S6**). The relative relations among these species and estimated time of divergence were summarized according to Jin et al. (2020); McPartland (2018); Murakami (2000); Murakami et al. (2006); Padgitt-Cobb et al. (2023). Note that the presence of HSR1 and HJSR in *C*. *sativa* suggests a shared pool of satellite DNA sequences originating from their last common ancestor.

In *Cannabis sativa*, HJSR is found mainly in subtelomeric regions, although, some chromosomes lack the HJSR signal (**Figure 1D**). The distribution of the HJSR satellite in *H*. *lupulus* was identical to that of the HSR1 satellite, including its localization on the sex chromosomes (**Figure 1D**; **Supplementary Figure S4D**). In *Humulus japonicus*, the HJSR satellite is present in subtelomeric regions, except for one arm of X chromosome, p-arm of chromosome 5, and both arms of chromosome 7, which carry 45S rDNA. No HJSR signal was observed on the Y_2_ chromosome (**Figure 1D**). The distribution of 45S rDNA across all analyzed species aligns with previous studies. The distribution of CS-1, HSR1 and HJSR satellites in female plants are shown in **Supplementary Figure S4**. We observed no sex-specific differences in the chromosomal distribution of these satellites between studied accessions (**Figure 1**; **Supplementary Figure S4**). However, the number of HSR1 signals varied in *C*. *sativa* (**Supplementary Figures S4C**, **S6**). The overall comparative distribution of CS-1, HSR1, and HJSR satellite further suggests intergenomic changes during the species divergence (**Figure 2**).

### 3.3 Characterization and origin of major satellites in Cannabaceae

**Figure 3:**
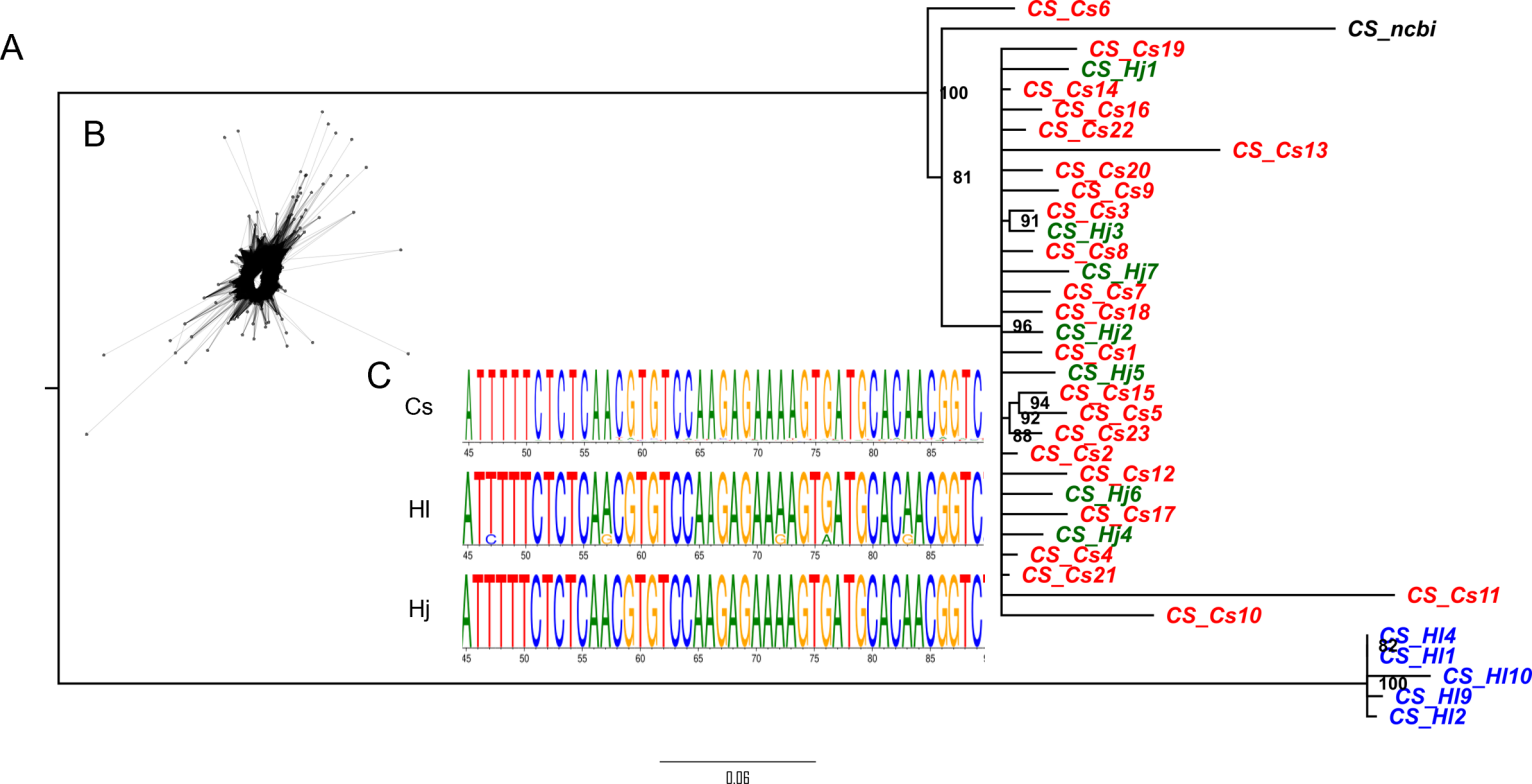
**(A)** The phylogenetic relationships of CS-1 sequences from *C. sativa*, *H. lupulus*, and *H. japonicus*. Notably, sequences from *H. upulus* form a distinct clade, representing stronger divergence compared to HSR1 or HJSR (**Supplementary Figures S8** and **S9**). CS_ncbi corresponds to the reference sequence available in GenBank under accession number JX402748.2. **(B)** Graph layout of CS-1 sequence. **(C)** Example of a conserved region within the CS-1 sequence shared among *C. sativa*, *H. lupulus*, and *H. japonicus*.

Using the primers sets designed for each major satellite across the three species, we obtained consistent amplification pattern for HSR1 and HJSR, with monomer unit length of approximately 380 bp. In contrast, the CS-1 satellite exhibited a species-specific ladder pattern in *H*. *japonicus*. The expected monomer band of 370 bp for CS-1 was obtained exclusively in *C*. *sativa* and *H. lupulus* (**Supplementary Figure S2**). Selected units for each satellite and species were cloned and sequenced. We analyzed a total of 35 CS-1 sequences (23 from *C*. *sativa*, 5 from *H*. *lupulus*, and 7 from *H*. *japonicus*), 46 HSR1 sequences (15 from *C*. *sativa*, 14 from *H*. *lupulus*, and 17 from *H*. *japonicus*), and 36 HJSR sequences (11 from *C*. *sativa*, 14 from *H*. *lupulus*, and 11 from *H*. *japonicus*). A comparison of satellite sequences cloned from each species supported low interspecific variability and their classification into one supercluster family, in line with physical localization on metaphase chromosomes (**Supplementary Figure S7**). To determine the relationships among these satellites in studied species, we conducted a phylogenetic analysis of individual sequence clusters (**Figure 3**; **Supplementary Figures S8**, **S9**). The CS-1 amplified in *C*. *sativa* and *H*. *japonicus* grouped together, while those amplified in *H*. *lupulus* formed a separate clade (**Figure 3**). Although the sequences from *C*. *sativa* and *H*. *japonicus* are closely related, no visible CS-1 signal was detected on the chromosomes of *H*. *japonicus* (**Figure 1B**), suggesting a low enrichment of these satellite units insufficient for detection by FISH. CS-1 was clearly localized to the subtelomeric regions of *C*. *sativa* chromosomes, in contrast to its (peri)centromeric localization on chromosome 2 in *H*. *lupulus*. The HSR1 and HJSR sequences tended to cluster together, with no unique species-specific clades identified between the species (**Supplementary Figures S8**, **S9**), indicating low divergence among individual subunits. This is consistent with their shared localization in subtelomeric regions. In *Cannabis sativa*, HSR1 was found in pericentromeric regions on five autosome pairs (**Figure 1C**). Among the three satellite arrays, only the HJSR satellite exhibited a conserved subtelomeric localization across all three species (**Figure 1D**), lacking clear species-specific clade and suggesting common origin for all three species.

## 4 Discussion

Satellite DNA plays a crucial role in the organization of plant chromosomes and has critical implications for evolutionary, genetic, and taxonomic research. Analyzing repetitive DNA divergence enables the exploration of evolutionary relationships among plant species (Yoong Lim et al., 2006; Koo et al., 2011). Despite the progress in characterizing the satDNA content within *Humulus* species, the organization, location and the extent to which these sequences are shared or diverged among the related species of the Cannabaceae family remain unknown. In this study, we examined the molecular and cytogenetic characteristics and phylogenetic relationships of major satellites CS-1, HSR1, and HJSR in *C*. *sativa*, *H*. *lupulus*, and *H*. *japonicus*. According to the library hypothesis, all three studied species should share a library of different conserved satellite DNA units (different satellite DNA families, monomer variants, and subfamilies within a satellite DNA family). The satDNA sequences may be differentially amplified in each taxon with the subsequent replacement of one sequence variant by another in different species or populations (Plohl et al., 2012). Similarly, the evolution of the HSR1 and HJSR satellites is likely to be explained by such a process. The presence of both satellites in *C*. *sativa* suggests that they originated from a common ancestor. It is tempting to speculate that their presence predated the speciation and split of *C*. *sativa* and *H*. *lupulus*. Although the number of repeat sequences varies significantly between species, differences in subcluster abundance likely result from species-specific amplification processes (Plohl et al., 2008, 2012). In contrast, the CS-1 satellite appears more diverse than the HSR and HJSR satellites (**Figure 3**). Correspondingly, the satellite RAE180 in *Rumex acetosa* has undergone distinct pattern of accumulation on autosomes and sex chromosomes. It is supposed that RAE180 originated before the split between *Rumex* species with XX/XY and XX/XY_1_Y_2_ sex chromosome systems (Cuñado et al., 2007; Navajas-Pérez et al., 2009b, 2009a; Quesada Del Bosque et al., 2011). Since *Rumex* species contain so far, the highest number of satellites on the sex chromosomes in plants (Jesionek et al., 2021), satellite RAE180 was hypothesized to be present in an ancestral genome in various monomer variants from which novel tandem arrays could later be amplified (Navajas-Pérez et al., 2009a). Such divergence was assessed in *R*. *hastatulus* Texas and North Carolina cytotypes, *R*. *acetosella* and *R*. *acetosa* (Navajas-Pérez et al., 2009a; Quesada Del Bosque et al., 2011), supporting again library hypothesis as shown for major satellites in this study, namely HSJR which shares similar chromosomal position and do not display specific sequence clustering (**Supplementary Figures S9**).

Although we confirmed the presence of CS-1 in the *H*. *japonicus* genome, this satellite was not detected on the metaphase chromosome using standard FISH approach, even with low stringency and higher sensitivity. We suggest that the absence of CS-1 positive loci on *H*. *japonicus* chromosomes rather indicate its low abundance in the genome and low tandem organization, similar to the RAE180 satellite in *R*. *acetosella* (Cuñado et al., 2007). These findings are consistent with the large phylogenetic distance between *Cannabis* and *Humulus* genera and suggest distinct evolutionary trajectories for satellites in the lineages leading to *H. japonicus*. SatDNA evolves rapidly through unequal crossing-over, replication slippage or mutation (Plohl et al., 2012). Amplification of satDNA may accompany karyotype rearrangements in plant species and chromosomal position of some satDNA family varies between related species (Navajas-Pérez et al., 2009a). In our study, we observed that the subtelomeric localization of the three major satellites was not conserved and showed the distinct distribution pattern across the studied species, except HJSR. Nevertheless, the interspecific differences were evident in chromosomal positions and number of foci of all three satellites, with predominant subtelomeric localization. The *Humulus lupulus* HSR1 satellite was localized to the (peri)centromeric regions of *C*. *sativa*. Similarly, CS-1 was found exclusively in the (peri)centromeric region of two autosomes in *H*. *lupulus* (summarized in **Figure 2**; **Table 1**). This satellite localization (**Figure 1C**) supports the hypothesis of large-scale genomic reorganization during the evolution in *Humulus* (Akagi et al., 2025). Further, this pattern is comparable to Cl12 in XY and XYY cytotypes of *R*. *hastatulus*, which during karyotype rearrangement was relocated from the (peri)centromeric regions and accumulated in subtelomeric regions of some autosomes and Y1 chromosome (Sacchi et al., 2024). Despite chromosomal rearrangements, including autosome and X-autosome fusions that occurred during the evolution in *H*. *japonicus* (Akagi et al., 2025), leading to a reduced chromosome number and the formation of X and both Y chromosomes, telomeric signals were consistently observed only at the terminal regions of all chromosomes.

In summary, the analysis of the major satellites in the Cannabaceae family reveals a complex evolutionary pattern during the speciation of *H*. *lupulus* and *H*. *japonicus*. Despite differences in abundance, the sequence structure of CS-1, HSR1, and HJSR remains largely conserved, we propose that their chromosomal localization has contributed to species-specific sequence evolution. This divergence may have played a role in speciation promotion, as evidenced by the distinct positioning of CS-1 and HSR1 arrays in *C*. *sativa* and *H*. *lupulus*. With the availability of long-read sequencing data, a more comprehensive analysis of the full organizational structure of these major satellites will be possible, facilitating the identification of new sequence motifs even among various cultivars and varieties within the Cannabaceae family.

## Data Availability

The datasets for this study can be found in the repository European Nucleotide Archive (ENA) under the accession number PRJEB81858. Raw data is freely available in the Zenodo data repository doi: 10.5281/zenodo.16672322.
