# Supplementary material for: Contrasting pattern of subtelomeric satellites in the Cannabaceae family
Source: Front Plant Sci. 2025 Aug 19;16:1631369. doi: 10.3389/fpls.2025.1631369 (PMC12406709; doi:10.3389/fpls.2025.1631369)
Supplement: Supplementary file 1 [file DataSheet1.docx]

Supplementary Material

# Supplementary Figures and Tables

## Supplementary Tables

| Primer name | Orientation | Sequence (3´- 5´) | GenBank Accession | Reference |
| --- | --- | --- | --- | --- |
| OPJ9 | Forward | ACAGAGTACAACTCAGAAACAAACC | KX688593.1 | Polley et al. 1997 |
|  | Reverse | AAGGTCGCACAATGACCG |  |  |
| MADC2 | Forward | GTGACGTAGGTAGAGTTGAA |  | Mandolino et al. 1999 |
|  | Reverse | GTGACGTAGGCTATGAGAG |  |  |
| HSR1 | Forward | CCCTCTGGTGAATTGGAGAT | PRJEB81858 |  |
|  | Reverse | CCTTTCAGAAATCTTCGATTTCTCTA |  |  |
| HJSR | Forward | GCGATGAGCTTACTATTTCTTCCA | PRJEB81858 |  |
|  | Reverse | CGATGGGATAACCGAAGAATTGG |  |  |
| CS-1 | Forward | GGTACCACTATGAGAAATGTGAG | PRJEB81858 |  |
|  | Reverse | CCTTTGTGAAATGTGGCCCGG |  |  |
| CS-1 (for *H*. *japonicus*) | Forward | GGTACCACTATGAGAAATGTGAGA |  | This study |
|  | Reverse | AAATGTGGCCCGGACTCATC |  |  |
| 45S rDNA | Forward | TGCCCGTTGCTCTGATGATT | AF223066.1 |  |
|  | Reverse | TCCACCAACTAAGAACGGCC |  |  |
| 5S rDNA | Forward | GTTTTCAGGGGTGCAACACG | MN537579 | Easterling et al., 2020 |
|  | Reverse | CTTACGGCTCAAAAGTTTGT |  |  |
| Telomere | Forward | TTTAGGGTTTAGGGTTTAGGGTTTAGGGTTTAGGG |  | Ljdo et al. 1991 |
|  | Reverse | CCCTAAACCCTAAACCCTAAACCCTAAACCCTAAA |  |  |

**Table S1** Sequences of primers used for PCR amplification and preparation of DNA probes for FISH analysis.

| **Hybridization mixture**  **Stringency** | 77% | 68% |
| --- | --- | --- |
|  | **Volume** (µl) | **Volume** (µl) |
| **100% formamide** | 10 | 7 |
| **50% dextran sulphate** | 4 | 4 |
| **20x SSC** | 2 | 2 |
| **HSR1/HJSR/CS-1** | 50 ng/µl | 50 ng/µl |
| **45S/5S rDNA** | 50 ng/µl | 50 ng/µl |
| **Water** | 2 | 5 |
| **Total volume** | 20 | 20 |

**Table S2** The composition of hybridization mixture for selected stringency conditions used in this study.

| **Cl5 (*Cannabis sativa*) – CS-1** | **Graph layout** |
| --- | --- |
| Consensus length: 370 bp  Proportion: 3.7 % | 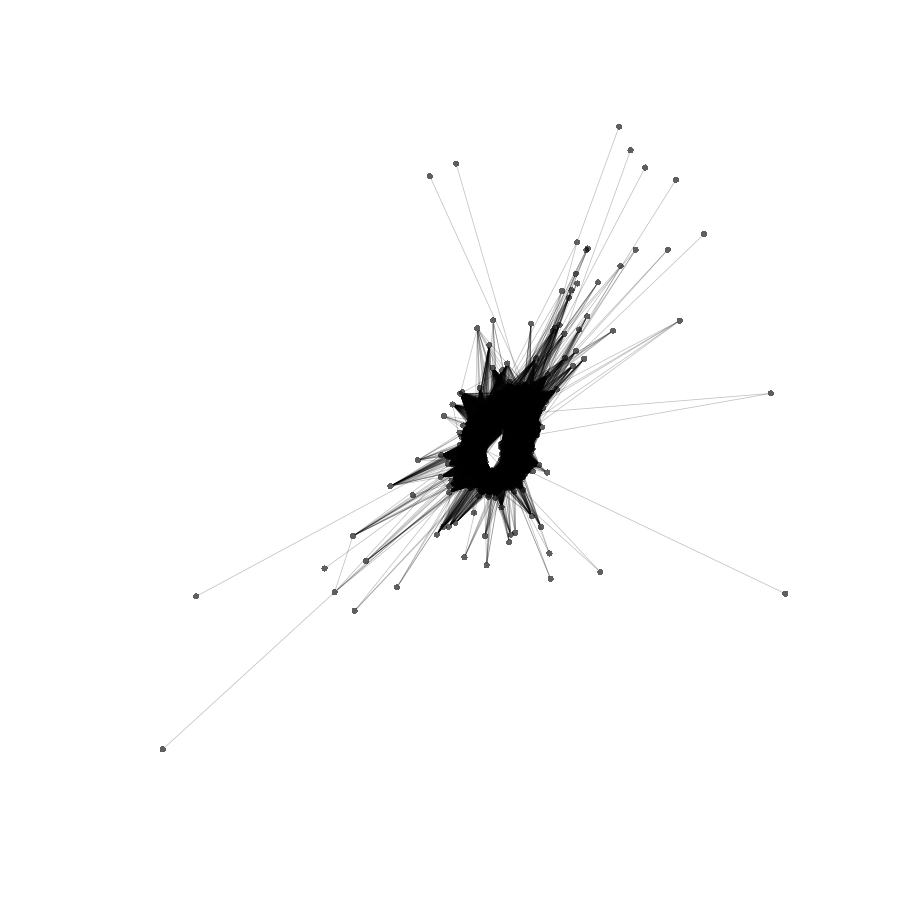 |
| TAGCCAAGACCGTTGTGCATCACTTTTCTCTTGGACACGTTGAGAGAAAAATTAGTTTTCTTGCACTAACGATCGTTTTAATCGAAATAGTGAAAATCTCACATTTCTGATAGTGGTACCCCTTTGTGAAATGTGGCCCGGACCAATCTCGGAATATTTTTTGCCACTAGTCACAATCAATTTGTACTACTAAGAAATGAATATGAAGAATGGTTTTGGGTTAAAATTTGTTGAAATGGCCCCCTAATTGAAAATATTCTTCTTTTCAGGGGAAATTTTAACAAGTTTTTTCCTTACTCTTACACTCGTTAGTTATCTGTTAAAATCTCAACCTACACATCTCCAAAGATCATGAAATTTCACGGGGGCC |  |
| **Cl75 (*Humulus lupulus*) – HSR1** | **Graph layout** |
| Consensus length: 383 bp  Proportion: 0.3 % | 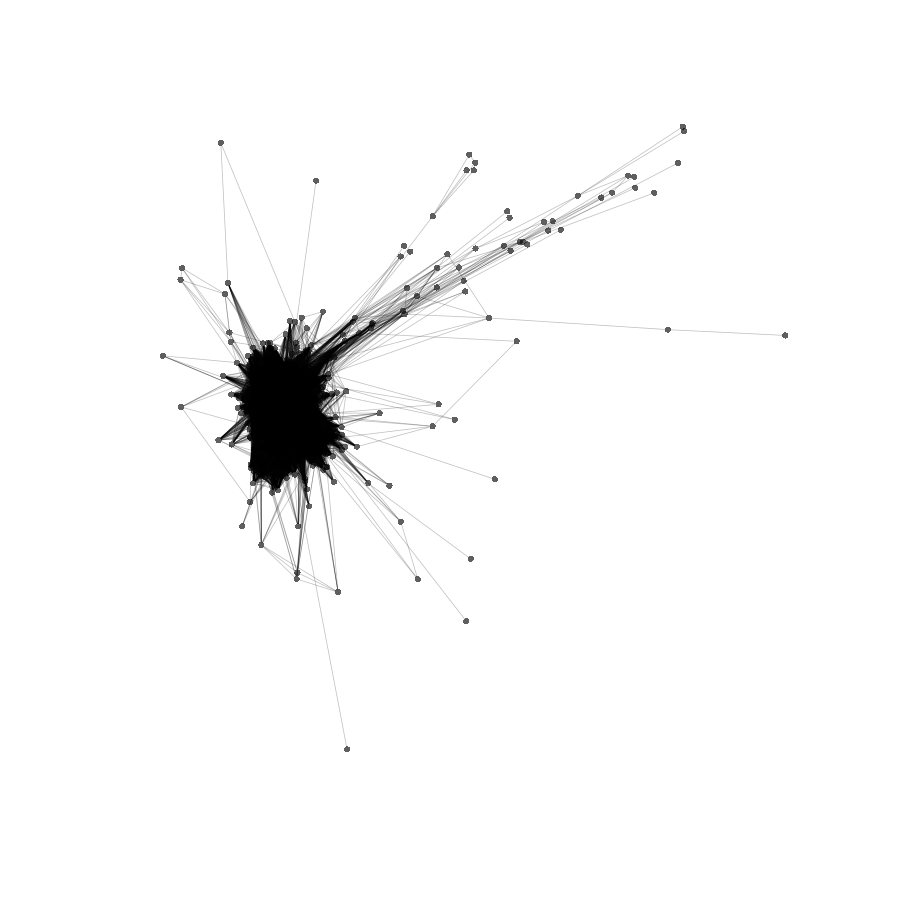 |
| TTTATACATAGAACCCTAAGACATAGGGCTACAACTTTTGTGCCCATGACCCTCGCATATTCTTTAGTATTCACTATCAAAACATTGCTCGAATCCATATTAGAGAAATCGAAGATTTCTGAAAGGGGTACCCCTTTGGTGAATTGGAGATTTCACTAATTTCTCCGAAATGTATTACCCTCGTGTTCAACTCGTTCGATATTTTTCCCCAAGAGATCTCATGGGTAAATGAATATATTGGAGTCGAAACGGAGCCTTAAGTCAACATTTCAAAAAAAATTCCCCTTTTTGATGGCAATTTTCGCAAGTTTTTTCCTTATATTCGTACATCTCTCAGTATTTTGACTGTATCACCCTCTACACACCTCGGATTTGCACGAAAT |  |
| **Cl57 (*Humulus japonicus*) – HJSR** | **Graph layout** |
| Consensus length: 380 bp  Proportion: 1.2 % | 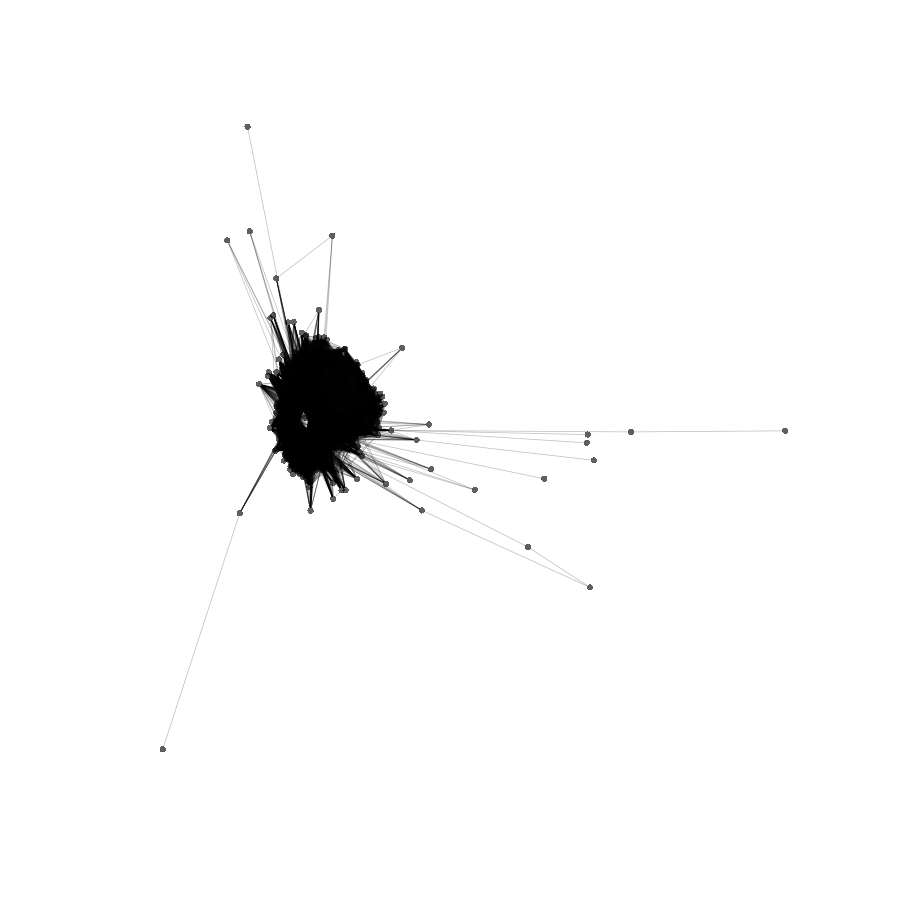 |
| ATTTTGTTGGTAGATGGCTAAGATATAGGGCTACAACTTTAAAAGAGGGCAAAACAACCAATTCTTCGGTTATCCCATCGAAAAAATAGCTCGAAGTTAGAGTTTGAAAATTGGAATTTTCGGAAAGGGGTACCCCTTTGGTGATTGAAGCTTAACTATTTCTTCCAAAAGTTTGACATAAGTGTGTCAAACACCAAAGATACTTATTAGGGTTACTTTTTATAGGGAAATGAGTCTCTTAGAGAAGTATCGAAGGCTAAACCTTCCGACAAAAAAAATTCGATTTTTCAAGGCAAAATTTCGCAAGTCTGGGGGCAATAGTAGCCCACGAGTCAGTATTTTGGTCATATCTCCCTCTATGCACCTCGGAATTGGTTGAA |  |

**Table S3** Major satellites identified in *Cannabis* and *Humulus* species using RepeatExplorer2 and TAREAN. For each satellite, the table includes the monomer unit length, consensus sequence, estimated genomic abundance based on sequencing data, and associated graph layout information generated during clustering analysis.

## Supplementary Figures


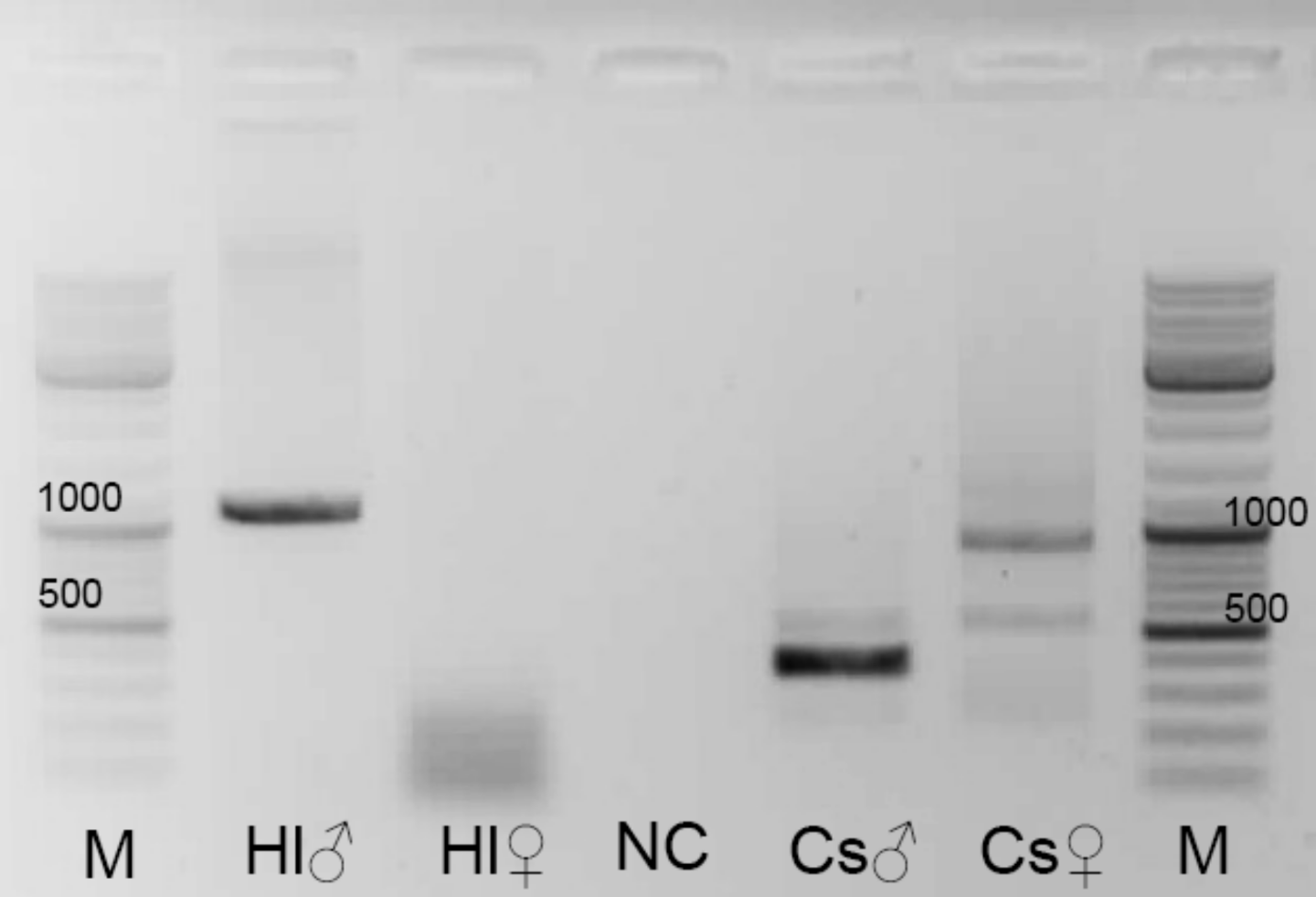


**Figure S1** The PCR-based sex determination in *Humulus lupulus* and *Cannabis sativa*. In *H*. *lupulus* male (Hl ♂), the OPJ9 primers amplified a male-specific region of approximately 1.15 kb, which was absent in the female *H*. *lupulus* (Hl ♀). Similarly, in *C*. *sativa* male (Cs **♂**), the male-associated MADC2 marker, with an estimated length of 390 bp, was clearly amplified only in male individual(s) and was not detected in *C*. *sativa* female (Cs♀). PCR products were separated in 1.5% agarose gel stained with ethidium bromide (EtBr). M – DNA ladder, NC – negative control.

**
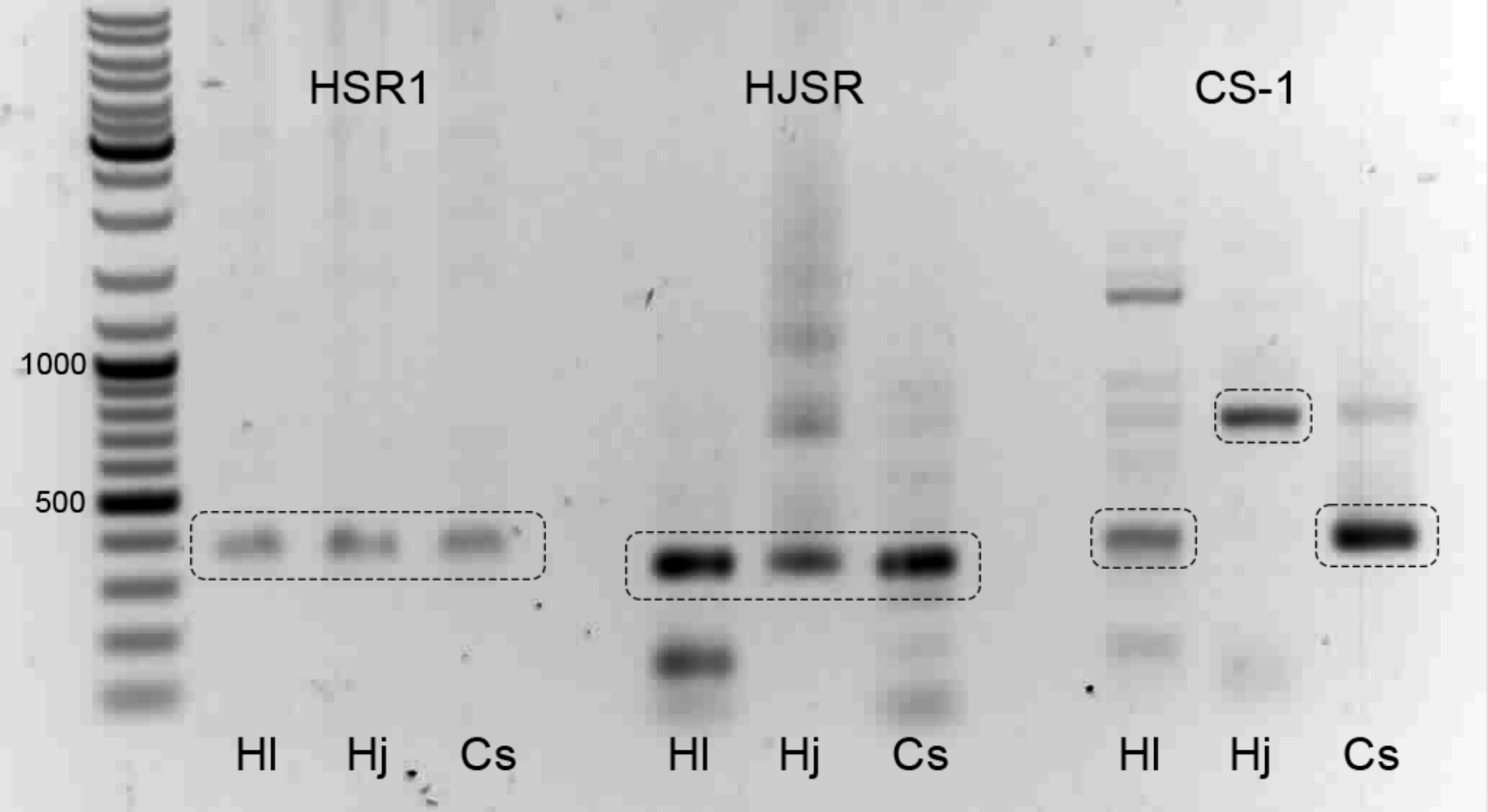
**

**Figure S2** PCR-based amplification of HSR1, HJSR and CS-1 major satellites. The patterns of HSR1 and HJSR are consistent in *H*. *lupulus* (Hl), *H*. *japonicus* (Hj), and *C*. *sativa* (Cs), using the same pair of primers. The CS-1 sequence exhibits a distinct pattern for each species. PCR products were separated in 1.5% agarose gel with EtBr staining. The red rectangles indicate selected DNA fragments, for each satellite and for each species.

**
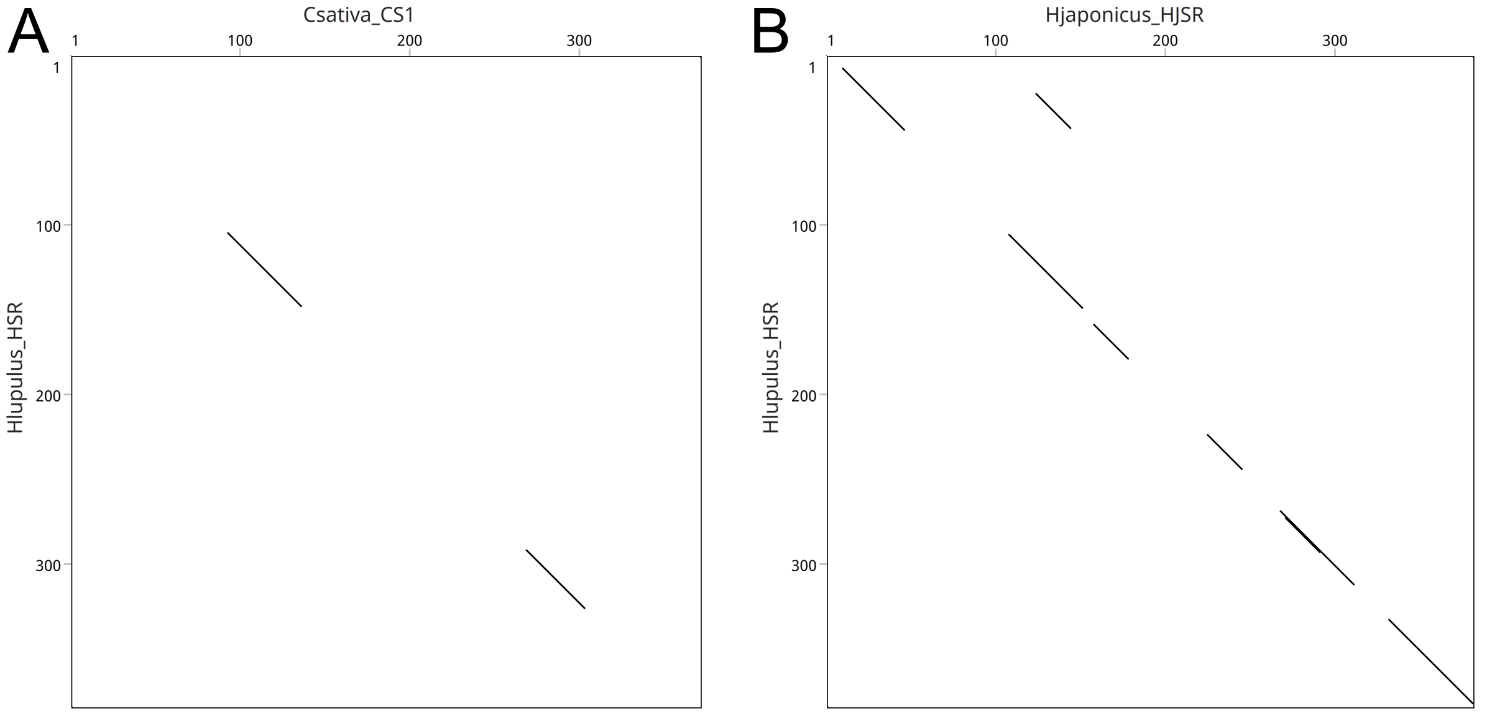
**

**Figure S3** Dot plot analysis of sequence similarity among the three studied satellites. The right panel displays the comparison between HSR1 and CS-1, showing 58.824% identity. The left panel shows the comparison between HSR1 and HJSR, with 65.051% sequence identity. As input files were used sequences in Supplementary Data Sheet 1.


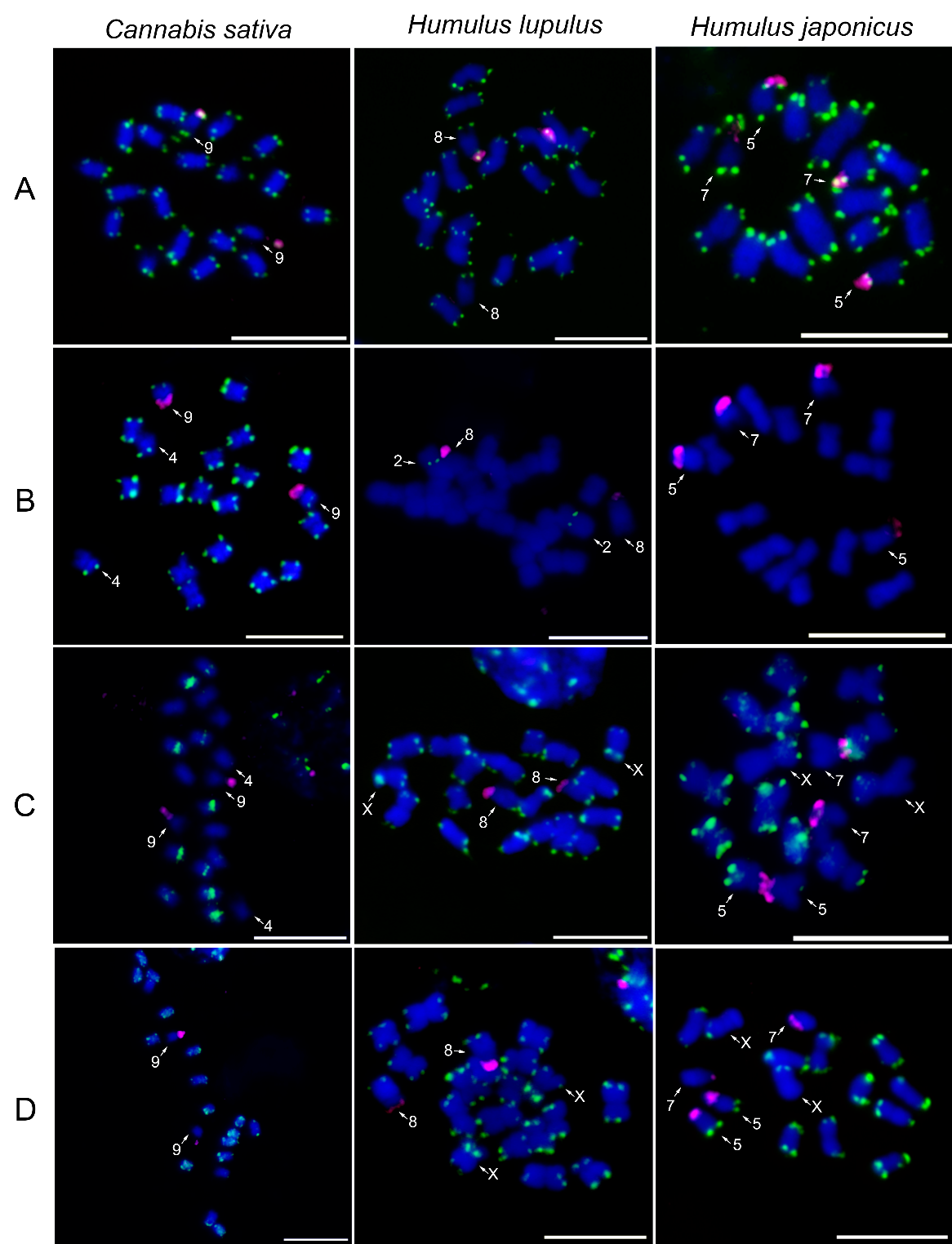


**Figure S4** Chromosomal distribution of major satellite in studied species. The localization of (A) telomeric sequence motifs (TTTAGGG, green), (B) CS-1 satellite (green), (C) HSR1 satellite (green), (D) HJSR satellite (green), and 45S rDNA (magenta) on female mitotic metaphase chromosomes of *Cannabis* *sativa*, *Humulus* *lupus*, and *H*. *japonicus*. Interestingly, HSR1 is localized within the (peri)centromeres in *C*. *sativa*, while displaying subtelomeric position in *H*. *lupulus* and *H*. *japonicus* (C). The identification and numbering of individual chromosomes follow the system previously established and described by Kim et al. (2008); Divashuk et al. (2011, 2014); Alexandrov et al. (2012); Akagi et al., (2025). Mitotic chromosomes were counterstained with DAPI. The arrows indicate differentiated autosomes and sex chromosomes. Scale bar = 10 µm.


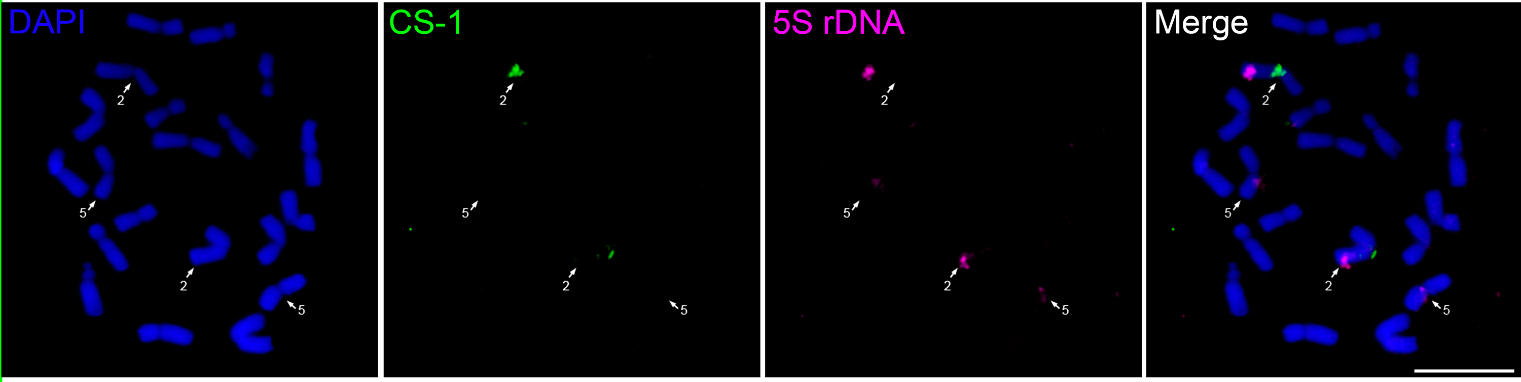


**Figure S5** Localization of CS-1 satellite repeat and 5S rDNA in male *Humulus lupulus*. The distribution of 5S rDNA (magenta) is consistent with the karyotype analysis described in Karlov et al. (2003). The 5S rDNA is localized in the subtelomeric region of chromosome 2 and the pericentromeric region of chromosome 5. Notably, both 5S rDNA and CS-1 satellite (green) are localized on chromosome 2. Mitotic chromosomes were counterstained with DAPI. Scale bar = 10 µm.


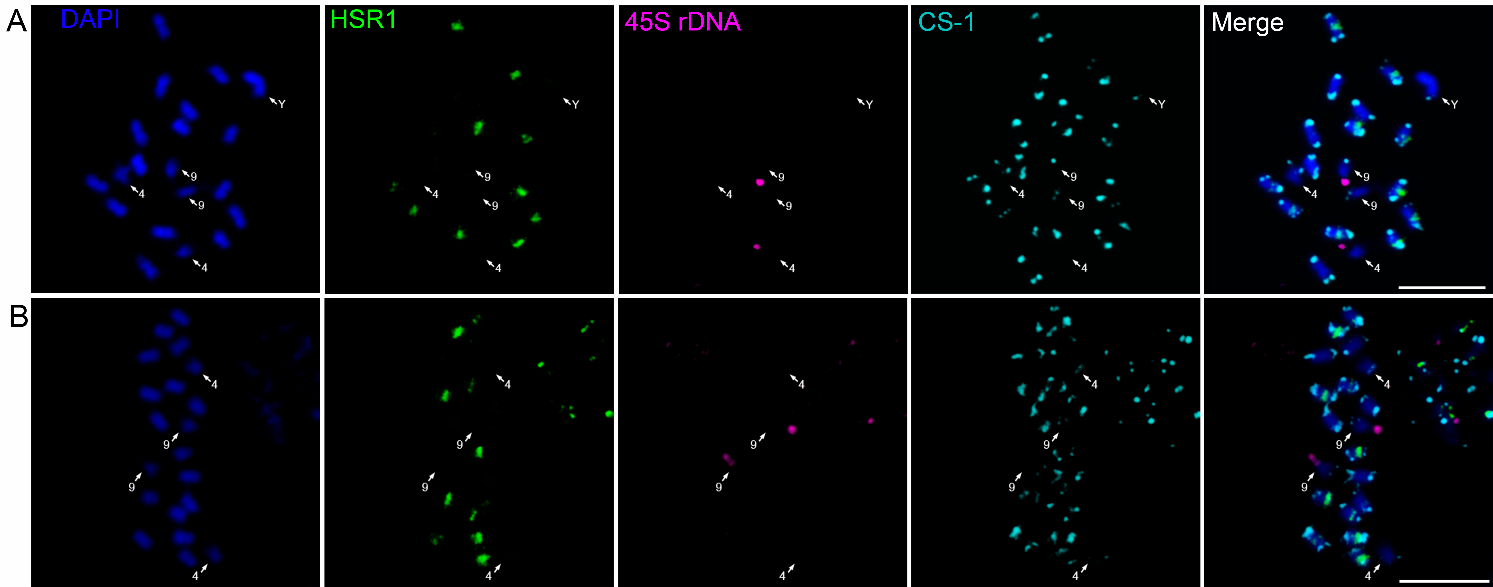


**Figure S6** The colocalization of selected satellite repeats in *C*. *sativa*. The distribution of HSR1 (green) and CS-1 (cyan) satellite repeats on metaphase chromosomes is shown for (A) male and (B) female *Cannabis* *sativa*. Ribosomal 45S rDNA (magenta) was used as a reference marker to identify chromosome 9. HSR1 exhibits (peri)centromeric signals on, the ten chromosomes in (A) male and on eleven chromosomes in (B) female. Mitotic chromosomes were counterstained with DAPI. Scale bar = 10 µm.


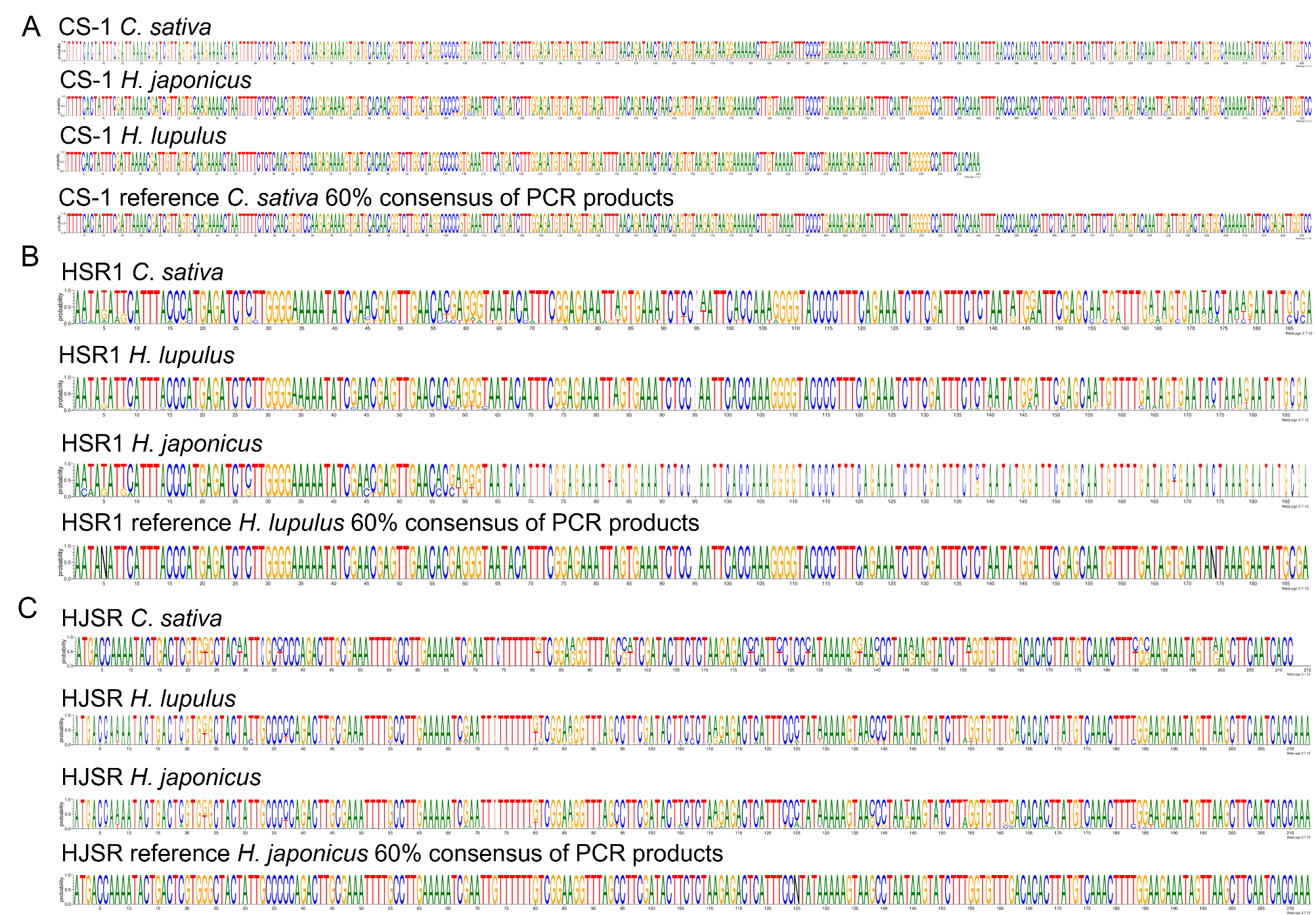


**Figure S7** Sequence logos and variability of major satellite repeats in studied species. The sequence variability of the major satellite repeats (A) CS-1, (B) HSR1, and (C) HJSR is shown for *Cannabis* *sativa*, *Humulus* *lupus*, and *H*. *japonicus*. Reference sequences were constructed from the consensus of cloned and isolated fragments, representing a 60% consensus sequence for each tandem repeat. The height of each letter in the sequence logo reflects its observed frequency at a particular position in the alignment. Notably, the length of the CS-1 repeat sequence varies among studied species, being overall shorter in *H*. *lupulus* than in *H*. *japonicus* and *C*. *sativa*.


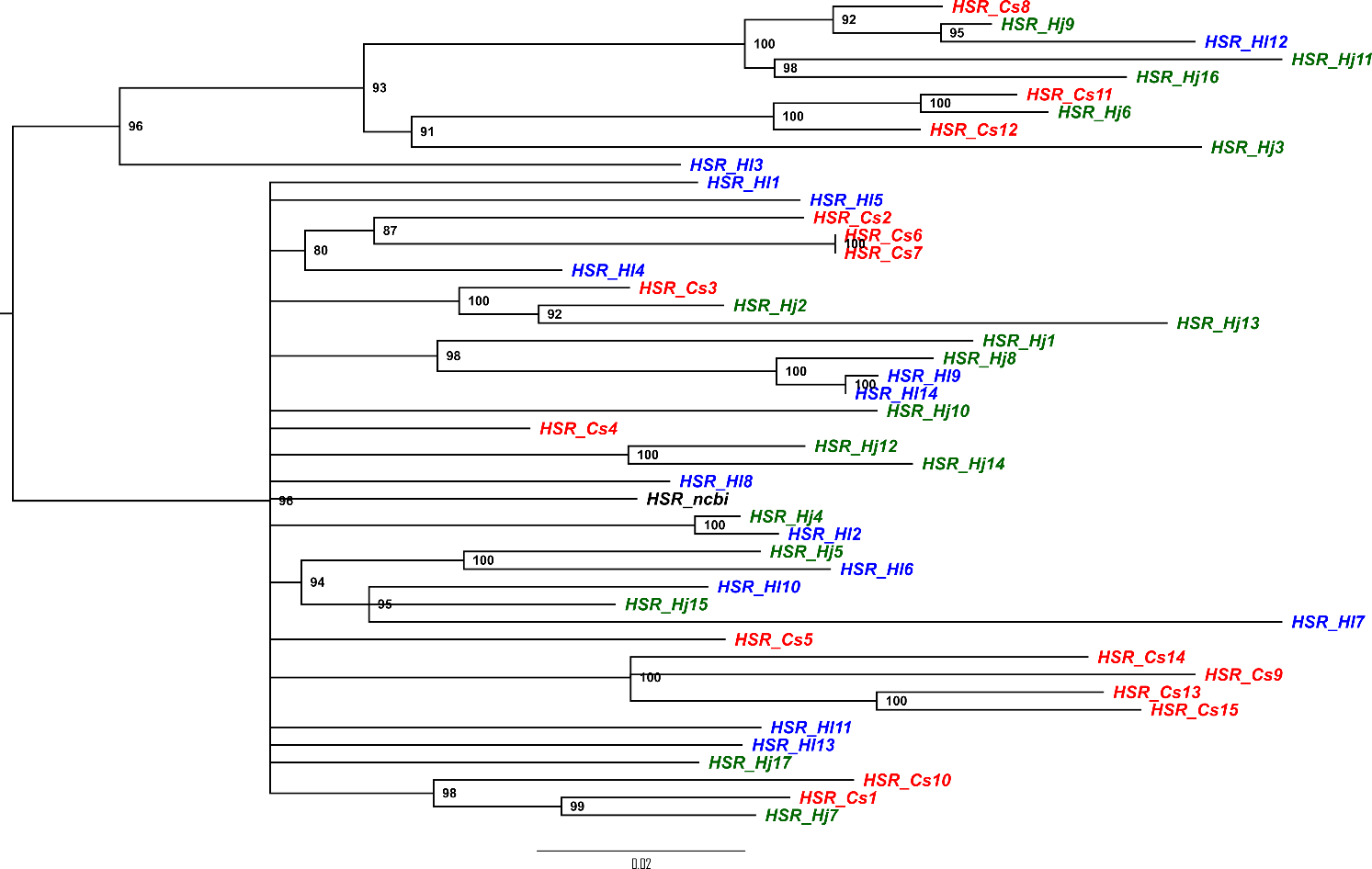


**Figure S8** Phylogenetic tree of HSR sequences from *C*. *sativa*, *H*. *lupulus*, and *H*. *japonicus*. The tree shows the relationships among HSR sequence variants across the three species. The distribution of sequence clusters on metaphase chromosomes either in pericentromeric or subtelomeric regions (see Figure 1 and Supplementary Figure S4), together with overall sequence similarity (Supplementary Figure S7), supports a lack of species-specific clustering in the rooted phylogenetic tree, supporting low sequence divergence for analyzed satellite cluster. HSR_ncbi corresponds to the reference sequence available in GenBank under accession number GU831574.1.


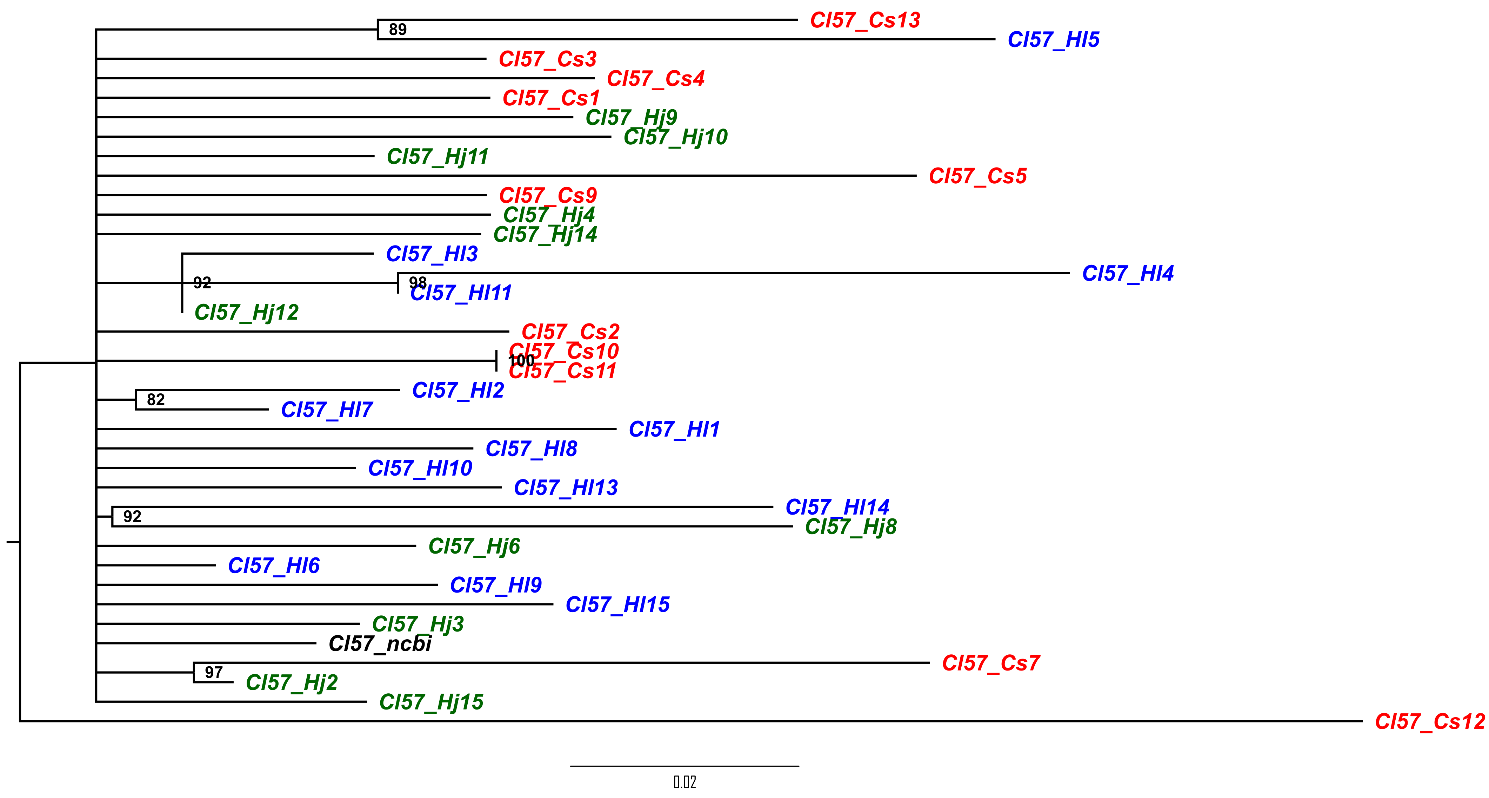


**Figure S9** Phylogenetic tree of HJSR (cl57) sequences from *C*. *sativa*, *H*. *lupulus*, and *H*. *japonicus*. The tree shows the relationships among HJSR sequence variants across the three species. The distribution of sequence clusters on metaphase chromosomes in subtelomeric regions (see Figure 1 and Supplementary Figure S4), together with overall sequence similarity for HJSR satellite (Supplementary Figure S7), supports a lack of species-specific clustering in the rooted phylogenetic tree, supporting low sequence divergence for analyzed satellite cluster. Cl57_ncbi corresponds to the reference sequence available in GenBank under accession number GU831573.1.
