# Supplementary material for: Contrasting pattern of subtelomeric satellites in the Cannabaceae family
Source: Front Plant Sci. 2025 Aug 19;16:1631369. doi: 10.3389/fpls.2025.1631369 (PMC12406709; doi:10.3389/fpls.2025.1631369)
Supplement: Supplementary file 2 [file Table1.docx]

>CS-1 consensus sequence

TAGCCAAGACCGTTGTGCATCACTTTTCTCTTGGACACGTTGAGAGAAAAATTAGTTTTCTTGCACTAACGATCGTTTTAATCGAAATAGTGAAAATCTCACATTTCTGATAGTGGTACCCCTTTGTGAAATGTGGCCCGGACCAATCTCGGAATATTTTTTGCCACTAGTCACAATCAATTTGTACTACTAAGAAATGAATATGAAGAATGGTTTTGGGTTAAAATTTGTTGAAATGGCCCCCTAATTGAAAATATTCTTCTTTTCAGGGGAAATTTTAACAAGTTTTTTCCTTACTCTTACACTCGTTAGTTATCTGTTAAAATCTCAACCTACACATCTCCAAAGATCATGAAATTTCACGGGGGCC

>HSR consensus sequence

TTTATACATAGAACCCTAAGACATAGGGCTACAACTTTTGTGCCCATGACCCTCGCATATTCTTTAGTATTCACTATCAAAACATTGCTCGAATCCATATTAGAGAAATCGAAGATTTCTGAAAGGGGTACCCCTTTGGTGAATTGGAGATTTCACTAATTTCTCCGAAATGTATTACCCTCGTGTTCAACTCGTTCGATATTTTTCCCCAAGAGATCTCATGGGTAAATGAATATATTGGAGTCGAAACGGAGCCTTAAGTCAACATTTCAAAAAAAATTCCCCTTTTTGATGGCAATTTTCGCAAGTTTTTTCCTTATATTCGTACATCTCTCAGTATTTTGACTGTATCACCCTCTACACACCTCGGATTTGCACGAAAT

>HJSR consensus sequence

ATTTTGTTGGTAGATGGCTAAGATATAGGGCTACAACTTTAAAAGAGGGCAAAACAACCAATTCTTCGGTTATCCCATCGAAAAAATAGCTCGAAGTTAGAGTTTGAAAATTGGAATTTTCGGAAAGGGGTACCCCTTTGGTGATTGAAGCTTAACTATTTCTTCCAAAAGTTTGACATAAGTGTGTCAAACACCAAAGATACTTATTAGGGTTACTTTTTATAGGGAAATGAGTCTCTTAGAGAAGTATCGAAGGCTAAACCTTCCGACAAAAAAAATTCGATTTTTCAAGGCAAAATTTCGCAAGTCTGGGGGCAATAGTAGCCCACGAGTCAGTATTTTGGTCATATCTCCCTCTATGCACCTCGGAATTGGTTGAA
